# Monstrosity

**Published:** 1855-05

**Authors:** 


*Monstrosity.* By G. P. Bœrstler, M. D., of Lancaster.

Dr. Dawson: In compliance with my promise, I now give you a history of the highly interesting and, as far as I have had the means of reference, altogether *unique* monstrosity.

On Tuesday evening, 16th January, I visited the wife of one of our most respected citizens, who was in her ninth labor; the uterine contractions strong, and at 15 minutes intervals. At 7 o'clock I touched, and found the os high up the sacrum, and dilated to a dime's size; 7½ o'clock—touched again, os dilating rapidly; 8 o'clock revealed it fully dilated, and the membranes slightly protruding; this examination gave the impression that a knee or elbow presented, and I at once placed the mother in the usual position for turning; I could not satisfy my mind as to the presenting part, and, therefore, ruptured the membranes, and then felt what I believed a groin, the hip resting in the left illiac region, the body oblique across the maternal pelvis. Into this *supposed* groin I hooked two fingers, and with some little traction brought the breech into the proper axis of the pelvis, congratulating myself on an ordinary breech presentation which would give me little trouble. Waiting to see whether the fœtus would now advance, and finding this not so, after some very vigorous throes, I again introduced the left hand for the purpose of bringing down the inferior extremities; and, feeling the supposed groin, I slipped up the hand to the knee, and in attempting to flex this I was foiled; continuing the examination of this limb, I felt the spinal column along and parallel to it; reflection could bring to my mind no such presentation, and partly withdrawing the hand to search for the fœtal pelvis, I found the two lower extremities flexed on its abdomen, both of which I brought down, unfortunately separating the left femur from its epiphysis. Having now three legs to deal with, the idea of twins was naturally fixed in my mind. The uterine contractions being vigorous, and the tractile force applied greater than in ordinary breech cases, I believed the leg of the other child to be the impeding obstacle, and at once introduced the hand to correct the entangled posture of the *twins;* carefully feeling this third leg, I found it firmly united to the body of the child whose two legs I had brought down, and arising from the dorsum of illium. A malformation was now evident, its character unknown. Hooking two fingers above the attachment of this back leg, and grasping the two other thighs with the right hand, I acted with considerable force on these two points, and found the pelvis and body of the child gradually advancing through the inferior strait, until it reached the junction of two bodies united by the intergrowth of the ensiform cartilages; at this point I met the greatest resistance during the labor; this being happily overcome, I found two bodies diverging from each other, which, however, passed the strait without much trouble; and by passing the hand up, I felt two apparently well formed heads, each as large as that of an ordinary child. To my mind it seemed impossible that those two heads could pass the strait at once, and I looked to the probable necessity of lessening one or both; but, to my great gratification, they passed without any extraordinary efforts being called for. This could not have resulted but for th *good fortune* of the head of the smaller child resting upon the neck an cheek, up to the malar bone, of the larger.

Thus was completed a delivery of a most extraordinary "lusus naturæ," to the well being of the mother and children.

The mother's recovery was rapid. The children are living—one vigorous, the other feeble—both take food, and urinate and defecate. There was one ordinary placenta and one cord.

*Description of the Children.*—The heads, faces, arms and hands, and the chest down to the ensiform cartilages, are well developed and in proper proportions. From the junction downwards, one body, its anterior surface from side to side broader than in an ordinary child—the abdominal muscles well developed—the umbilicus in situ—two spinal columns perfect, the coccyx of each terminating on each side of the anus, and about half an inch from it—two pelves *united*, the left one belonging to the larger child, encroaching upon and lessening that of the right side. The sex female, one vulva and one anus, the opening of the latter not larger than an ordinary rye straw; the two lower extremities are of proper size and proportions.

Upon the back, and from the dorsum of the two illia, arises a leg, running up between the two spines and the two inner shoulders, and terminating in a right and left foot, joined at the heel. This is, in truth, a double leg, enveloped in one common integument, having two femurs, two tibias and two fibulas; the leg admits of partially moving it directly backwards, to a distance of several inches from the back of the child.

I have no doubt that nature designed one of these legs for each child; for when the right child is awake it moves its lower extremity, as also the left foot of the double leg; when asleep the limb is quiet—and so of the other child. Tickling the sole of either foot, movement follows in the limb, but I have not perceived motion in the opposite leg to the one tickled.

The two bodies have each its heart and lungs—puerile respiration distinct—the action of each heart easily felt, though the first and second sounds can not be distinguished, owing to the rapid systolic and diastolic movements.

So far as I have been able to detect, I believe the respiratory and cardiac action of both children to be synchronous, though the harmony of the former is interrupted by the crying of either child. I have frequently observed that when one of the children is nursing and the other crying, the latter falls to sleep; so frequent is this occurrence that I hold it to be the rule, and it shows the strong sympathetic relation between these *two distinct human beings joined in one.*

What the unison of organs may be in the abdominal cavity we, of course, have no means of knowing; all reasoning thereon must be hypothetical. That the viscera are duplicated, we think probable. In the feeble child there existed the anomaly of the frœnum linguæ, arising from the dorsum of the tongue about half an inch from the tip, and inserted into the palatine arch, of course rendering the tongue useless in sucking.

The following admeasurements were made in the presence and by the aid of Drs. Effinger, Davis and Wagenhals. Owing to the want of calipers the circumference was taken. Occipito Frontal, $13\frac{1}{4}$ in. in the larger, $12\frac{1}{2}$ in the smaller; Bi-parietal, 6 in., $5\frac{1}{2}$ in.; this is half the circumference, having measured from one parietal protuberance to the opposite, across the vault. Mental, $4\frac{3}{4}$ in., $3\frac{3}{4}$ in. Occip.: Bregmatic, $13\frac{1}{2}$ in., $13\frac{1}{4}$ in.; Shoulders, 10 in., 9 in.; Junction of Bodies, $16\frac{1}{4}$ in.; Pelvic, $13\frac{1}{2}$ in; length from head to foot, $17\frac{1}{2}$ in., 17 in.; weight, 10 pounds.

These are the facts in this, to me, exceedingly interesting case, and I very much regret the absence of men fully capable to examine it in all the lights which science demands of its votaries.

In the development of Embryos in Utero, certain starting points are always *essential* to the production of particular organs or limbs. This law is clearly manifested in the production of the double leg, for in the fusion of the two pelves the *acetabular portion* is presented, and hence the development of this extra limb.

A letter from Prof. Meigs refers me to the great work of M. Serris on Monstrosities—to it I have no access, but from the professor's letter, I learn that a specimen approaching mine was born in Sardinia a few years ago, and brought to Paris for public exhibition; the union in this case was in the two pelves, and called by M. Serris a *pelvidym.* In mine the union is from the Zyphoid cartilages down, and, hence, I ask if it is not properly called a Hepato-pelvidym?

*Death of the Children.*—On Tuesday morning, 20th February, the mother observed the larger gasp a few times, and at 15 minutes before 8 o'clock, breathing ceased; at 8½ o'clock I saw it, and could not detect any respiratory act, nor pulsation of heart or arteries. Drs. Effinger and Wagenhals pointed out to me the apparent movements of the carotids. We, however, all became satisfied that *these movements depended upon the circulation of the smaller child.* The asphyxiated condition continued for *four and a half hours—no respiration, no pulse, the capillaries of the skin filled with dark blood*, giving a purple hue to its entire body, and *beautifully showing* the *demarkation* between *the asphyxiated* and *living child;* this line, from the junction down, extended half an inch to the right side of the umbilicus. A violent effort in coughing by the smaller child communicated a shock to the larger, convulsive movements of its limbs followed, and it uttered a few feeble cries, when it again relapsed into its condition of suspended animation, and so remained till five o'clock in the evening, when the smaller child died—one gasp in the larger, and in 10 seconds it slept with its sister. Thus these children fortunately survived their unfortunate union only five weeks.

Accompanying this history you will have daguerreotypes of the front and back views. These pictures are fac-similes of the original, and executed by my fellow-townsman, V. M. Griswold, in his style of accuracy and finish. You have seen the children, and I ask you to add to the above any remarks you may think proper.

---

*Remarks* by Richard Gundry, M. D.

It will not be deemed inappropriate, or improper, to append to the foregoing interesting account of Dr. Bœrstler, of the unique malformation it was his fortune to meet with, condensed accounts of some other malformations which have occurred from time to time. Our readers, however, will remember that it is by no means intended in the few sentences we shall bring together, to furnish a complete essay on the subject; for neither the short time allowed to us, nor the sources of information within our reach, would enable us to do more than specify a very few of the more remarkable malformations which have occurred, and to add but a few observations which seem necessary in the premises.

The cases already observed have been sufficiently numerous to have been classified under several divisions. These have varied with the author that introduced them, each new observer adding a classification as a modification, and, in his estimation, an improvement upon those of his predecessors. Without entering into too much detail, suffice it to say that they may all be reduced to three great varieties founded upon deviations from the normal standard in—(I) *Position;* (II) *Form;* and (III) in *Number* and *Size* of organs.

I. Malformations *from the wrong position* of proper organs, constitute, perhaps, the most numerous class. The variations in the course of blood-vessels, familiar to every anatomist, the special terror of the operative surgeon, are the most common instances. In one case, the right subclabian artery arising from the left side of the arch of the aorta, and passing between the œsophagus and trachea, gave rise to such difficulty in swallowing that the subject, (a female,) could hardly summon resolution enough to force down sufficient food to prevent starvation. The same anomalies in the position of muscles are also not unfrequently observed. Even the viscera follow the same course occcasionally. Dr. Baillie describes a case in the Philosophical Transactions, in which the organs within the chest and abdomen were transposed so that the heart was placed on the *right* side and the position of its cavities and vessels was reversed. The liver occupied the *left* hypochondriac region, and the spleen the *right.* The great end of the stomach was seen to the right of the abdomen, and the pyloric orifice a little on the left of the spine. The mesentery was inclined obliquely from right to left. The illeum terminated in the great intestine on the left side, and the cœcum was to the left of the psoas magnus and ilicacus internus muscles. The arch of the colon passed from the left to the right side of the body, and the sigmoid flexure crossed over the right psoas muscle to get into the pelvic cavity. The vessels and nerves in relation with these parts were normal in their size, but corresponding in their relations to the altered situations of the viscera. During life nothing unusual had been suspected.

A soldier aged 72, died, and was examined by Mery, when the same transpositions were observed in the chest and abdomen.

Mr. Abernethy describes a case of a child ten months old, where the viscera, but not the blood-vessels, were transposed.

Among the anatomical preparations belonging to the Starling Medical College in this city, is a dried preparation of a man in which the heart is found on the right side, the liver on the left, and the internal viscera generally reversed.

II. Next to the malformations arising from changes of position of organs, those in the *form* are the more numerous. In these cases the number of organs, and even their position, may be normal, but their form is altered so as to constitute a deformity. They may resemble similar organs belonging to animals—may consist in an improper division of parts which should be united, or in the fusion of organs usually separate. Fœtuses, resembling the fabled Cyclopes, are occasionally met with where a coalescence seems to have taken place between the eyes. There are many varieties of this deformity which is comparatively frequent among animals, and especially among swine. They are usually born alive, but are not viable. So union of the lower extremities, with more or less atrophy, may take place, forming one apparent leg with two feet; or only one whole limb, or even only an imperfectly shaped caudiform mass. They are not viable.

There are other amalgamations frequently met with which do not influence the viability of the infant, such as coalescence of fingers or toes, which may be partial, having separate bones, but one envelope of soft parts, or having blended bones and soft parts. Various of the viscera which occurs in pairs, may sometimes be blended as the kidneys and ovaries.

The most common instances of cleavage of organs are the different forms of hare-lip and cleft palate. Fissure of the tongue—of the vertebræ, constituting spina—bifida—of the thorax and abdomen—of the bladder, with or without inversion of the mucous membrane—of the urethra—of the diaphragm, giving rise to congenital diaphragmatic hernia—of the scrotum forming many instances of so-called hermaphroditism, have been recorded by many sound observers.

III. We come now to the last form of monstrosity arising from deviations in the *number* and *size* of organs in which there is either a deficiency or excess. Almost every one of the organs has been found wanting in individual cases. Acephalous fœtuses are by no means rare. It has fallen to the lot of many practitioners to bring them into the world. In these either the whole head or some of its components may be absent. Thus the bones of the cranium or face may be wanting, or some portion of the nervous centres, the brain and spinal marrow.

These cases, though usually still-born, are sometimes viable. They have lived, cried and suckled many hours. In one case of congenital absence of the bones of the cranium, but where an imperfect cerebrum seemed to exist, the child survived six days. In other respects it was perfectly formed. It took no food, and had no evacuation of any kind. Respiration went on naturally; it did not cry, but often made a hideous, whining noise. When the soft substance at the top of the head was touched, general and violent convulsions took place. No signs of voluntary motion were observed. The spinal marrow may be absent with or without the brain. In one case recorded, the spinous processes were all absent, exposing the spinal canal the whole length; there was no spinal chord, but its place was supplied by a vascular membrane. Most of these fœtuses, according to Sœmmering and Morgagni, are females. Portions of the face are absent, but more often in animals than in man.

The trunk may be deficient in several respects. The neck may be short from absence of the cervical vertebræ, giving an appearance to the head as of a cat's head, which name (katzen kopfe) has been applied to them in some parts of Germany. Internal organs are absent in some cases. Sir Benjamin Brodie met with a fœtus of nearly the natural form and size in which there was no heart. There was no communication between the trunks of the arteries and veins. The vena cava of the fœtus was continued so as to form the umbilical vein, and the internal iliac artery of one side was reflected to form the umbilical artery. There were various other defects, as in the fingers and toes. The liver was deficient, and the palate cleft. Several other cases are mentioned in the Memoirs of the Academy of Sciences in Paris for 1720 and 1740. Many other instances are recorded by modern observers, especially one by Dr. Clarke, in which the brain and heart were both absent.

The extremities may be absent, one or all, or atrophied. Fingers and toes are often absent, and even limbs. Individuals born without certain limbs, have survived to a good age, and astonished the world by their exhibitions of the manner in which they perform with their organs processes usually belonging to those absent. A child who had no ear or any perforation for one, and yet heard, is mentioned. Animals are born without certain limbs, of which Haller gives many instances, among others a cat without fore legs; and at this time, at any rate during the past summer, one of the attractions at the museum and gardens on the Canada side of Niagara Falls, consisted in a dog born without fore legs, which seemed at that time lively and healthy in all other respects.

The reproductive organs may be severally absent, especially one testis or an ovary; nor does this appear to affect the propagation of the species. The uterus has been known to be rudimentary—even absent. The penis still more often.

In this class also may be included those cases of deficient opening, as atresia ani—vaginæ—oris—where cloacal terminations therefore exist.

Malformations, with one or more supernumerary organs, are comparatively frequent. Geoffrey St. Hilliaire gives an instance of seven fingers on one hand, and eight toes on one foot. Similar cases are recorded by other authors. Those born with six fingers are by no means rare, and once formed, the peculiarity is liable to be perpetuated to succeeding generations.

Much more rare than these are those congenital malformations where a number of organs are added, so that an approach to duplication is discovered with more or less ease. The duplication may be so nearly complete that the monster appears as the union of two children, as in the case described by Dr. Bœrstler*, or one of them being so atrophied appears as the parasite of the more perfectly developed child, to which it is united. These are termed monsters by implantation, and the former "double monsters," or by coalition.

All varieties of supernumerary parts have been observed from the addition of a single muscle or vessel, internal or external organ, and limb, to a complete human being, from those having a single finger added, to the coalition of two perfect bodies, as in the Siamese Twins. An additional head has been observed often in animals. An ox is mentioned in the Philosophical Transactions with an extra head attached under the lower jaw, and a cow attained its full growth with two heads and necks.* Such anomalies form the staple wonders of many traveling showmen. Rarely this deformity has been observed in man. Sir Everard Home's case is interesting in this relation. It was an Indian child with a double head, which lived to the age of two years, and died then, from the bite of a *Cobra di Capello.* The midwife, horrified at the strange sight of a double head, endeavored to destroy the infant directly after birth by throwing it on the fire, where it remained long enough to burn one of the eyes and ears to some extent. The body was naturally formed, but the head double. Beside the proper head another of the same size, apparently perfect in shape, was attached at the upper part. They were attached at the crowns, the upper head being inverted. They were firmly united, without any indentation. The face of the upper head was not over that of the lower, but had an oblique position, the centre being directly over the right eye. The neck of the upper head was two inches long, and its extremity terminated in a soft tumor like a small peach. The eyes of both heads moved at the same time, but those of the upper head in different directions. The sides of the upper contracted on exposure to light, its eye-lids were never completely closed, but remained a little open, even when the child was asleep. Tears flowed almost constantly from them, but never from the eyes of the lower head, except in crying. The superior head seemed to sympathize with the child in most of its natural actions. When it nursed, the upper head seemed to express satisfaction by an increased flow of saliva from the mouth, though it always flowed a little from it. Both faces smiled. When the skin of the superior head was pinched it did not feel as much as if the lower were touched. No pulsation could be detected in the situation of the temporal arteries of the upper head at the age of six months, but the superficial veins were very evident. The head is now in the Hunterian Museum in London. The two skulls are nearly of the same size, and equally ossified. The frontal and parietal bones, instead of being continued over the top of the head, meet each other, and are united by a circular suture. The two skulls are almost equally perfect at their union, but the superior skull, as it recedes from the other, becomes imperfect, and many of its parts are deficient. They both have the same number of teeth. No septem of bone divides the two crania so that the two brains must have been contained in one long case.

An Italian was seen in 1698 with another head than his own proper one, connected to the chest below the cartilage of the third rib. It adhered by the lower half of the right side of the face and head, so that the right ear and surrounding parts were not seen. All the rest was plainly discernable. The man felt whatever touched the additional head.

Other parts of the body may also be additional. The vertebræ may be increased in number, and give rise to a caudal extremity. Men with imperfect tails have been observed by competent authorities. Indeed, if we may credit certain travelers' stories of late years, this, which has been considered a very rare anomaly, must be raised above the class of deformities, and described as one of the characteristics of a race,—of which, doubtless, more proof than has yet been given will be required by this skeptical age!

An additional abdomen and lower extremities were observed by Winslow to be attached to the left side of the epigastric region of a girl 12 years old. The second body was small. It had a row of vertebræ connected to the sternum of the other. Both bodies discharged fœces. There were no muscles in the additional body, the interval between the bones and skin being filled with fat, blood vessels and nerves. Whatever touched the additional body was felt by the child. There were no sexual organs.

Bartholei and Zacchias relate that they saw with astonishment the double monster, who was exhibited in many of the principal cities of Europe, named Lazarus Colleredo. He was 28 years old, well formed and of the usual stature. To his chest was attached another deformed being, hanging from the lower end of the sternum. It had two arms with three fingers on each hand, and one imperfect lower extremity. The head was larger than that of Lazarus himself, but not well formed. It was covered with hair but no beard. The trunk seems to have been imperfect. The eyes were generally closed, the mouth open, with saliva constantly flowing from it. There was a pulsation in the chest. Respiration was hardly perceptible. The hands, ears and lips could be moved. It was nourished by food taken in by Lazarus. The pious parents had caused the addition to be christened separately from Lazarus, under the name of Johannes Babtista. On this point Zacchias raises an argument, as to whether John Babtista had a rational, distinct soul. As he inclines to the negative, he doubts the propriety of its baptism, but reverently bows to the decision of the church, in which he does not acquiesce. On the origin of these addenda he determines that they are

"*additamenta ex luxuriante semine enata, et quod nullam ne per somnium quidem rationalis animæ potentiam sortirentur.*"

In Ambrose Pare's works mention is also made of a man, forty years old, who had an additional body, perfect in its parts except the head. A similar deformity suspended to the navel of a boy, six years old, is recorded by Amatus Lusitanus. A Gentoo boy is mentioned in the Philosophical Transactions, who had a little brother suspended to the pubes, and consisting of pelvis and lower limbs. He felt whatever was done to this, but could not move the legs, which were cold.

Beside these, monsters formed by the addition of other supernumerary organs, there are some, where (with additional organs, more or less perfect) the points of union are unlike the organs of a natural body, but exhibit a coalescence of the same parts of the two bodies, in various degrees and shapes. In animals this is more often the case than in man, and a friend of the writer has a specimen of a young pig with two distinct bodies and a head formed apparently by the blending of two normal heads. In the work of Sœmmering, plates represent a series of such cases, from a head naturally formed to two natural heads joined, in which the intermediate grades are filled by individuals differing almost imperceptibly from each other. The coalition may take place at almost any point. In Rita Christina, born on the 12th of March, 1829, at Possari, in Sardinia, the head, neck, and upper extremities were doubled, while the chest, abdomen and lower extremities were more or less fused together. It lived about eight months, and died in Paris, whither it was taken for experimental purposes. An interesting case of this kind we shall notice after we have glanced at other varieties of malformations.

The thorax may be double and the union take place in the abdomen, as in a case of Forieps, born at Stamsried, in Bavaria, Jan. 1, 1838. Two children joined by the abdomen, double above and having one penis and two lower limbs, but no rectum, lived seven days and died within a quarter of an hour of each other. Another of the same character is said to have lived in Scotland during James IV.'s reign, for 28 years. One body died some days before the other. To the same category belong the celebrated Hungarian Sisters. The duplicature of organs was, however, more complete. They were born at Szony, in Hungary, in 1701, and died in 1723, when they were buried in St. Petersburgh. They were joined at the back, below the loins, and had their faces and bodies placed half sideways toward each other. They had one anus and one vulva. The viscera were all double, except that the two vaginæ united into one toward the external aperture, and the two recta terminated in the same way. There were two bladders and urethræ opening separately. The two sacra were blended into one and had a single os coccygis connected to the lower end. The two aortæ were joined into one tube before the division of the iliacs, and the inferior vena cava were united in the same place. They were not equally strong nor well made. They had separate wills, and the strongest dragged the other after her when she wanted to go any where. At six years old one had a paralytic affection, which left her much weaker than the other. They had different temperaments. Neither the alvine nor the renal evacuations were performed at the same time by both sisters. The menses happened at different periods, more than a week intervening. Sometimes one, sometimes the other, would be disordered at such periods. One slept oftentimes while the other was awake; one had a desire for food while the other did not. They suffered from small pox and measles at the same time, but other diseases attacked them separately. Judith was often convulsed, while Helen was well. One of them had a catarrh and colic without affecting the other. Their intellectual capacities differed. They were, however, both lively, merry and well bred, and could converse in several languages, as Hungarian, German, French and English. They died together.

To these instances of coalescence of organs and bodies we may add those where a body has contained another body, or individual parts, of which several examples might be quoted. We have only space for one instance of a *fœtus in fœtu*—which is described by Mr. Young, in the first volume of the Medico-Chirurgical Transactions. From the time of birth a tumor was perceived in the abdomen of the child, which increased until the time of death, which occurred at nine months. A firm and thick black cyst was placed in front of the abdominal aorta between the origin of the celiac and mesenteric arteries, attached to the left crus of the diaphragm, and covered in front by the stomach and duodenum, pancreas and its duct, and transverse portion of the colon. It contained 78 oz. of limpid fluid and a rudely formed human fœtus, adhering to its surface by a fleshy cone, proceeding from the umbilicus, and measuring one inch and seven-tenths at one end, and one inch at the other. This production was covered by integuments of the natural appearance, in which there was sebaceous matter. The extremities were distinctly recognizable, and in many respects well formed. There were distinct fingers, toes and nails, but very short and stout. There was something corresponding to the basis of the cranium—a large portion of the spine, some short ribs, sacrum and ossa innominata, and some bones and joints of the limbs, well formed. Very little muscular substance was found in this creature, none on the trunk, a little about the hips and none in the remainder of the limbs, which consisted of adipose substance. There was neither hair nor spinal marrow, but a distinct plexus of nerves just within the umbilicus, about the commencement of the intestines, to which numerous branches were distributed. There were two locks of hair just below the parts corresponding to the head. There was neither heart nor lungs, nor abdominal viscera except a few inches of naturally formed intestine with mesentery. Two kinds of vessels were distinguished, but their connection with the cyst was not made out. A scrotum and hairs were distinctly cognizable.

Not only may parts of two separate bodies be thus in various ways united, but monstrosities have been recorded in several instances in animals, and a few in man, where parts of three bodies entered into their formation. Thus, according to Geoffrey St. Hilliare, Drs. Reina and Galvagni observed in Catania in the year 1832, a child with three heads.

We translate the following case from Walter's Anatomical Observations,* which was examined and dissections of it made by Dr. Johann Gottlieb Walter, and do so the more fully as it more nearly resembles Dr. Bœrstler's unique case than any we have discovered in our limited researches.

Anna Maria Woblack, aged 35, residing near Berlin, was confined on the 17th November, 1773, of twins, consisting of a fine boy, who did well, and the following monster who died during labor.

*External Appearance.*—The monster appeared composed of two children, whose faces looked toward one another. They were joined at the breast by union of the zyphoid cartilages—the space, therefore, between the fœtuses

*Walter's Anatomische Beobachtungen, &c. Berlin. 1782.

above formed a triangle, with the apex below and the base by a line drawn between the heads. The heads are about the same size. One neck however, is shorter and more contracted than the other. The two arms of the same side are also smaller than of the other side. Otherwise they are equally well formed as those of ordinary children. The umbilical cord was attached to the middle region of the abdomen common to both children. The umbilical artery was larger than ordinary—the veins as usual. There were two proper lower extremities and a mal-formed middle leg with seven toes. Each child had its own sacrum on which the vertebral column rested, and from which a normal os innominatum proceeded on one side, the pubis of one child forming a symphisis with that of the other. On the other side, between the two sacra, was situated an abnormal ilium, or rather a bone, as of two ilia united, from the lower part of which hung the third lower extremity, attached by ligaments so that each child had a normal leg and proper pelvic bone, and a third ilium and deformed middle leg were common to both. This leg, on dissection, contained a femur, a tibia, thick and contracted, bent to a considerable angle about its middle—the usual bones of the foot, and seven bones of the metacarpus, six of which formed a row, and the seventh, which was the largest, supported two toes, united as by a firm web. The sex was male —there was a penis and scrotum, but no testes therein.

*Internal Appearance.*—There were found two pair of lungs, two sets of ribs and vertebræ, two sterna united at the cartilages, two hearts, the left being the smaller, abnormal in the position and the distribution of its great vessels. Those of the right side were normal. There were two livers blended somewhat anteriorly, two spleens, two stomachs, two duodena, two jejuna, and two ilea, which united at the latter end to form one canal, one colon, cœcum, and rectum, two kidneys in the left side, and one, very large, on the right. From these, ureters proceeded to the bladder common to both. The distribution of blood vessels was exceedingly anomalous in the cavity of the abdomen. The testicles had not descended into the scrotum.

Can these multiform departures from the normal body be attributed to any law governing their production, or to any accidental external influence. The ancients laid great stress on the power of the imagination of the pregnant woman, to which was attributed the production of all nœvi and other deformities. Of later days the fashion has been to decry all such hypotheses, yet to substitute no reliable explanation. That a severe nervous shock in the mother may exercise some power in the arrest of development of the fœtus is by no means improbable in some few instances, but there are cases where an over-development (so to speak), rather than an arrest of the process, seems to have taken place. Moreover, the cases of transposition of various organs remain to be accounted for. The contingencies which must all occur during pregnancy, in order to a proper healthful gestation, are as little known as the laws of development of fœtal life, and the absence of any of the former, which may vitiate the proper action of the latter, are subjects in the investigation of which but little progress has been made. By a careful collection of these anomalies, and other kindred subjects, it may be that some insight can be eventually gained into the mysterious laws to which the growth of the embryo is subject, and the accidents by which their operation is suspended or thwarted. At present it is far easier to negative the various theories put forth from time to time on the subject, than to suggest any proposition suitable to explain the existence of all—of even a majority—of the ascertained malformations.

